# Intraosseous Ganglion Spanning the Scaphoid and Lunate: A Case Report

**DOI:** 10.7759/cureus.56045

**Published:** 2024-03-12

**Authors:** Shunpei Hama, Masataka Yasuda

**Affiliations:** 1 Department of Orthopaedic Surgery, Yodogawa Christian Hospital, Osaka, JPN; 2 Department of Orthopaedic Surgery, Baba Memorial Hospital, Sakai, JPN

**Keywords:** temporary scapho-trapezoidal joint fixation, bone curettage, lunate, scaphoid, intraosseous ganglion

## Abstract

Intraosseous ganglions (IOGs) are actually quite common but one spanning two adjacent carpal bones is uncommon. We report a case with an IOG spanning the scaphoid and lunate, which was treated surgically. A 16-year-old right-handed female noticed left wrist pain that started spontaneously five years previously. Physical findings indicated carpal instability in the left wrist. Posteroanterior radiographs of the left wrist showed small cysts in the lunate and scaphoid, while the lateral radiograph revealed volar flexion of the lunate. Bone curettage was performed using sharp curettes, and due to the physical findings of carpal instability, temporary scapho-trapezoidal joint fixation was done using two Kirchner wires. Two years post-surgery, wrist pain had significantly improved and carpal instability findings disappeared. Computed tomography revealed no obvious collapse of carpal bones and expansion of bone defects in the lunate and scaphoid. Bone formation was observed in the bone curettage area of the scaphoid.

## Introduction

An intraosseous ganglion (IOG) of the carpal bones is not uncommon but usually asymptomatic. Crabbe first used the term "intraosseous ganglion" [1]. IOGs are most frequently located in the lunate [2], and they are typically asymptomatic, often identified incidentally on wrist radiographs. However, Schajowicz et al. reported that 60% of their IOG cases had pain, lasting from months to years [3]. An IOG spanning two bones is even rarer. In a cadaveric study, the prevalence of IOG was 9.6% [2]. The detected peak of IOG is in middle age, with 56% of the IOGs located at the palmar carpus. The lunate and scaphoid were more frequently affected than other carpal bones, including the metacarpals and phalanges [4]. IOG spanning both the lunate and scaphoid is uncommon. While there is one case report in Hebrew of an IOG existing in scaphoid and lunate bones [5], no such cases have been reported in English. We present a case with an IOG spanning scaphoid and lunate accompanied by carpal instability, treated surgically.

## Case presentation

**Figure 1:**
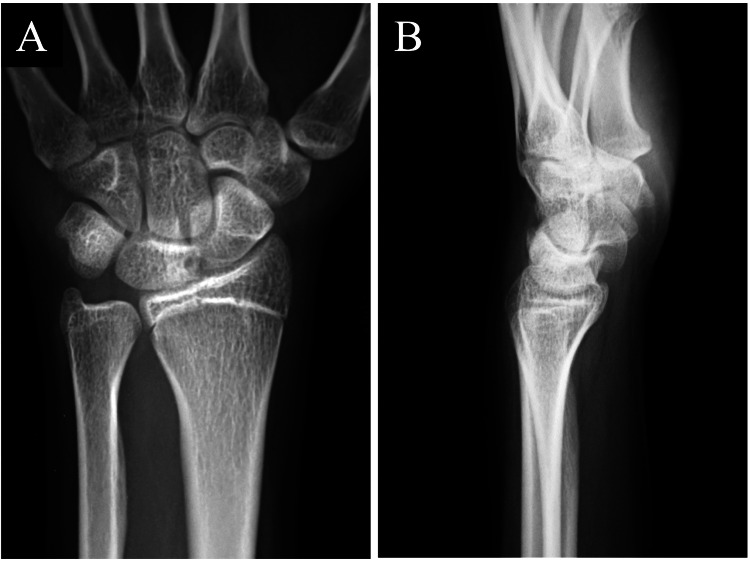
Preoperative radiographs of the left wrist. (A) Posteroanterior view. (B) Lateral view.

**Figure 2:**
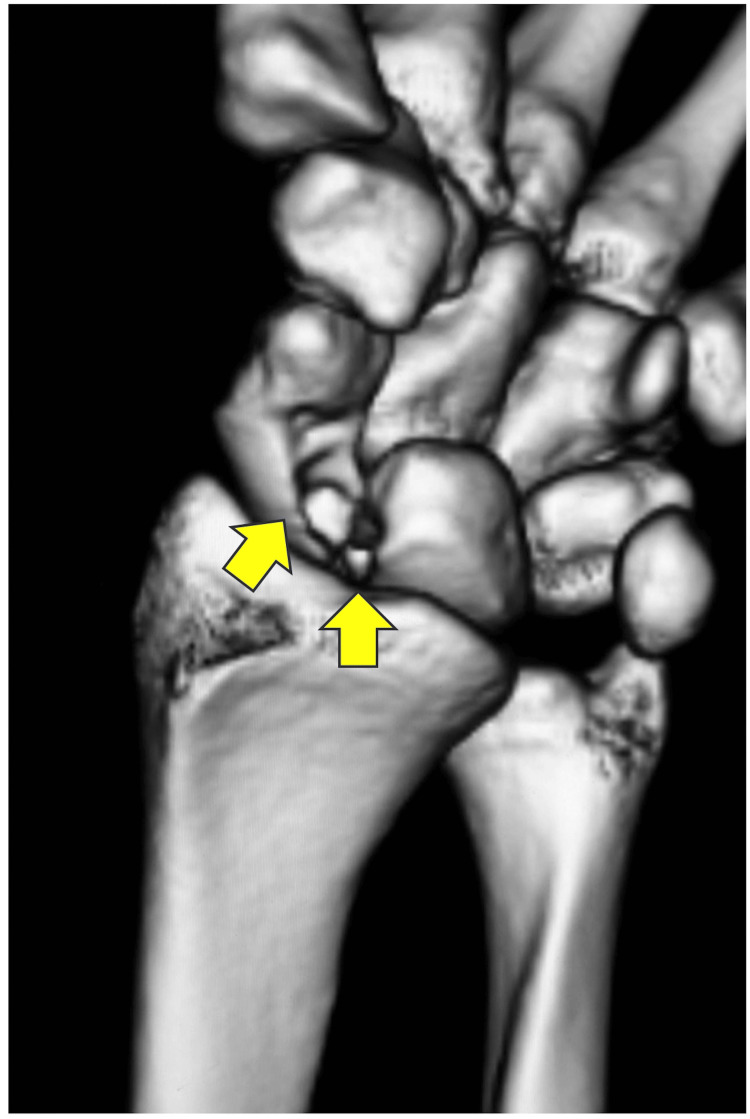
Preoperative three-dimensional computed tomography image of the left wrist (arrows: bony defects).

**Figure 3:**
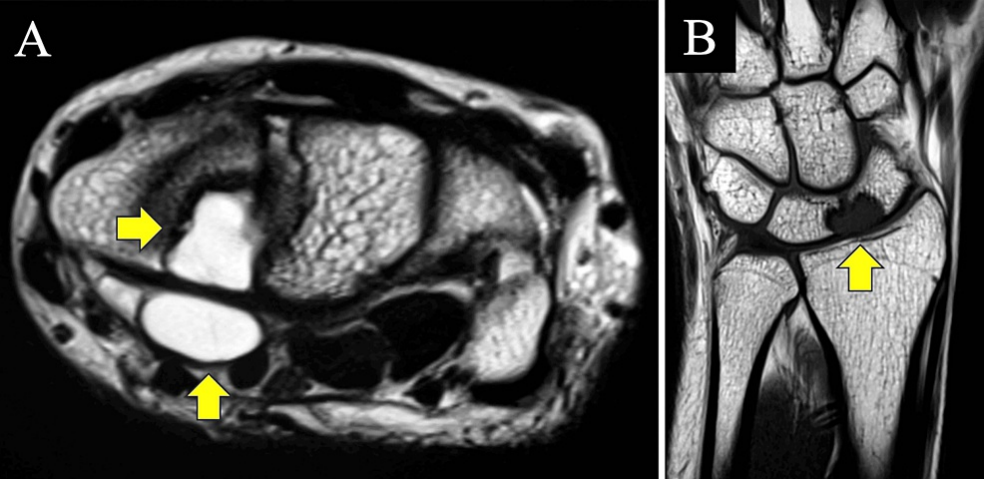
Preoperative magnetic resonance image (MRI) of the left wrist (arrows: ganglion). (A) Axial T2-weighted image of the MRI. (B) T1-weighted coronal image of the MRI.

A 16-year-old right-handed female presented with spontaneous onset pain in the wrist five years before presentation. She belonged to a dance club and aspired to become a dancer. Conservative treatments such as intra-articular steroid injections and wrist braces at other hospitals were ineffective, leading to her referral to our hospital. Her right and left grip strengths were 22 and 20 kg, respectively. We used a Jamar hydraulic dynamometer to measure her grip strength. The ranges of motion (ROMs) for right and left wrist extension were 85 and 65 degrees from neutral, respectively. Both ROMs for wrist flexion were 80 degrees. The dorsal wrist syndrome sign [6], scaphoid shift test (SST) [6], finger extension test [6], and articular non-articular (ANA) tenderness [6] were positive. These signs and tests indicated carpal instability [7], including scapholunate dissociation in the left wrist. The Disabilities of the Arm, Shoulder, and Hand (DASH) score was 29. A posteroanterior radiograph of the left wrist from two years ago showed small cysts in the lunate and the scaphoid. The lateral radiograph revealed slight volar flexion of the lunate (Figure 1). The radiograph of the wrist of the opposite site revealed a similar volar flexion of the lunate. Therefore, the volar flexion was not supposed to be pathologic. Bony defects were found on the scaphoid side of the lunate and the lunate side of the scaphoid in the computed tomography (CT) scan during the first visit to our hospital. Bony defects were also identified on the proximal palmar side of the scaphoid and lunate (Figure 2). Magnetic resonance imaging (MRI) of the left wrist showed a tumor, presumed to be a ganglion, in the proximal palmar side of the scaphoid and lunate, continuous with a subcutaneous ganglion. Its features were consistent with an IOG spanning adjacent sides of the lunate and scaphoid bones. Continuous preoperative MRI in another hospital revealed gradual tumor growth, prompting surgical planning (Figure 3). Because of these MRI changes and the carpal instability findings, surgery was planned.

**Figure 4:**
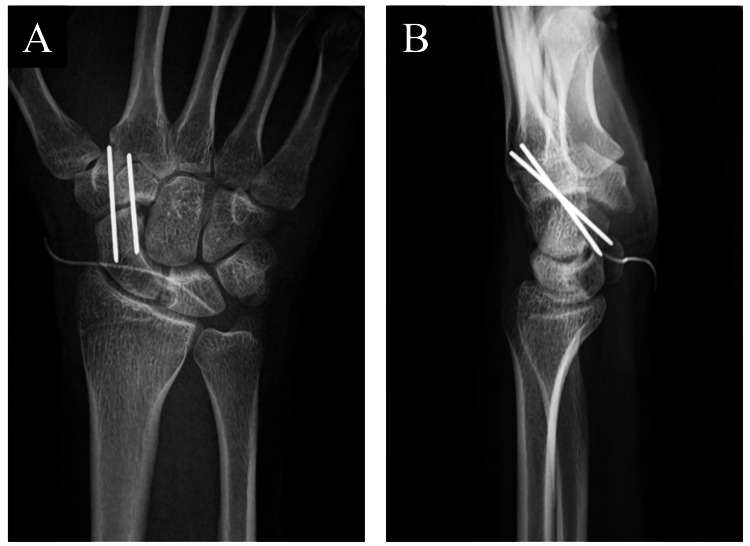
Postoperative radiographs of the left wrist. (A) Anteroposterior view. (B) Lateral view.

We performed the surgery under a microscope to clarify the deep operative field where the carpal bones are located. To minimize the operation scar, we made a transverse incision on the radiopalmar side of the radial styloid level. We shifted the flexor carpi radialis to the ulnar side and approached the palmar side of the scaphoid and lunate. The large subcutaneous ganglion was exposed proximal to the scaphoid. There was no obvious damage or degeneration of the volar scapholunate ligament, although it was not confirmed in detail. We dissected the ganglion from its subcutaneous location down to its osseous origin and excised it in total along with curettage of carpal bones. Bone curettage was performed on the bones with sharp curettes. Due to the physical findings of carpal instability, we performed temporary scapho-trapezoidal (ST) joint fixation using two Kirchner wires (Figure 4).

**Figure 5:**
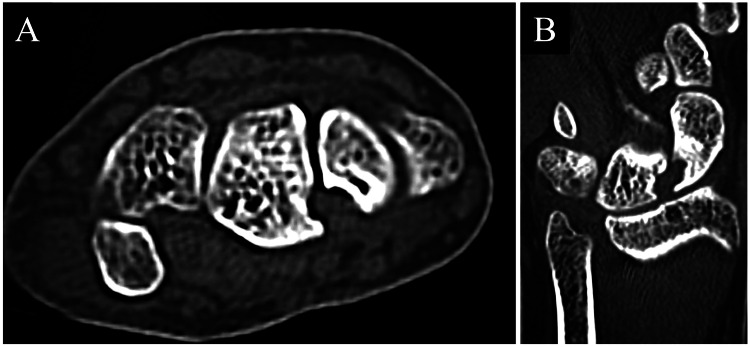
Computed tomography (CT) two years after the surgery. (A) Horizontal image of CT. (B) Coronal image of CT.

After the surgery, the wrist was immobilized with a cast for three weeks. After removing the cast, the patient wore a wrist orthosis for three weeks. We removed the Kirchner wires two months postoperatively and initiated active exercises. The patient was allowed to resume sports activities three months after the surgery. The wrist pain improved three to six months postoperatively. Two years postoperatively, wrist pain had significantly improved, and she was able to dance without difficulty. The outcomes at two years postoperatively were as follows: right and left grip strengths were 28 and 24 kg, respectively; ROMs for right and left wrist extension were 85 and 80 degrees, respectively; both ROMs for wrist flexion were 80 degrees. SST [6] and ANA [6] tenderness were negative, and carpal instability findings disappeared. The DASH score improved to 4.3. The CT revealed no obvious collapse of carpal bones and expansion of the lunate and scaphoid bone defect (Figure 5). There appeared to be bone formation in the bone curettage area of the scaphoid.

## Discussion

There was a predilection in the location of IOGs, with those in the lunate mostly located near the radiocarpal joint in the proximal part of the bone, and many IOGs in the lunate eroding toward the scaphoid. IOGs in the scaphoid were mainly located in the proximal portion near the lunate [2]. Uriburu et al. reported 15 cases of IOG that underwent surgery, with two of 13 patients having bilateral IOGs. Among them, six lunates and nine scaphoids were affected, and eight patients experienced wrist pain [4]. Three pathogeneses of IOG, including intramedullary metaplasia, penetration, and microvascular deterioration, were presented [2]. Bugnion found a close relationship between many bone cysts and periarticular capsular and ligamentous insertions [8]. In the present case, the mechanical stress or degeneration of the insertion of the radioscapholunate ligament to the scaphoid and lunate might be a cause of this ganglion. There are two types of IOG: type 1, idiopathic or primary intraosseous lesion, and type 2, the penetrating type caused by the cortical penetration of a previously existing soft tissue ganglion [4,9]. The causes of type 1 IOG remain unclear, while type 2 is caused by the invasion of proximal cortical material. The present case was considered type 2.

Due to the findings of carpal instability, we performed temporary ST joint fixation. Watson et al. performed permanent scaphotrapeziotrapezoid (STT) joint arthrodesis for adult Kienböck’s disease to avoid the rotary subluxation of the scaphoid leading to degenerative changes in the wrist [10]. Ando et al. employed a temporary ST joint fixation method for adolescent Kienböck’s disease using a few Kirchner wires and reported excellent clinical and radiographic outcomes [11]. Because the present case involved an adolescent with carpal instability findings, we opted for temporary ST joint fixation. Watson et al. classified carpal instability as static, dynamic, and predynamic [7]. In this case, we considered the patient to have predynamic carpal instability, which was demonstrable on physical examination but not by radiographic studies, and instead of STT joint fixation, we performed temporary ST joint fixation to prevent rotary subluxation of the scaphoid. However, the effectiveness of temporary fixation for the present case was not clearly established.

Surgical indications for IOG included cysts that progressively entered and replaced the cancellous substance of the cancellous bone or presented with a cortical defect. Bone cortical erosion and replacement might weaken the involved carpal bone, leading to a pathological fracture. In the present case, we believe the ganglion between the lunate and the scaphoid grew and penetrated these two carpal bones. Fearing that the ganglion might lead to scapholunate dissociation if left untreated, we performed bone curettage using the palmar approach whose advantage was the preservation of dorsal scapholunate ligament, which is stronger than the palmar part of the ligament and temporary ST joint fixation [11]. This case seemed to be IOG spanning an important anatomical location of the scapholunate ligament, which resulted in some carpal instability. This instability was only mild with no degenerative changes in the surrounding articular surfaces and treatment led to the resolution of the symptoms and restoration of function. Since the location of the ganglion was on the volar aspect, the effect on the carpal kinematics was minimal as the ligament is relatively weaker at this location. A dorsal location of a similar ganglion where the ligament is much stronger [12] might have resulted in greater instability.

## Conclusions

In conclusion, we performed bone curettage for an IOG existing in the scaphoid and lunate to prevent scapholunate dissociation, and temporary ST joint fixation was done using two Kirchner wires due to the physical findings of carpal instability. There were three key points of this case report. First, the ganglion gradually became larger and larger with conservative treatment and the patient presented with signs of carpal instability, the surgery was finally indicated. Second, the carpal instability could be caused by the bridging IOG if it was in the vicinity of an important ligament. Finally, an IOG spanning the scaphoid and the lunate was extremely rare. Our treatments led to the resolution of the symptoms and her wrist function recovered. The timing of the surgery in this case seemed to be appropriate. This was the first report of surgery for an IOG spanning two carpal bones written in English.
